# Preliminary study on diagnosis of gallbladder neoplastic polyps based on contrast-enhanced ultrasound and grey scale ultrasound radiomics

**DOI:** 10.3389/fonc.2024.1370010

**Published:** 2024-04-24

**Authors:** Zhengyi Qin, Jianmin Ding, Yaling Fu, Hongyu Zhou, Yandong Wang, Xiang Jing

**Affiliations:** ^1^ Department of Ultrasound, Tianjin Third Central Hospital, Tianjin, China; ^2^ Tianjin Key Laboratory of Extracorporeal Life Support for Critical Diseases, Tianjin, China; ^3^ Artificial Cell Engineering Technology Research Center, Tianjin, China; ^4^ Tianjin Institute of Hepatobiliary Disease, Tianjin Third Central Hospital, Tianjin, China

**Keywords:** radiomics, ultrasound, CEUS, gallbladder, nomogram

## Abstract

**Objective:**

Neoplastic gallbladder polyps (GPs), including adenomas and adenocarcinomas, are considered absolute indications for surgery; however, the distinction of neoplastic from non-neoplastic GPs on imaging is often challenging. This study thereby aimed to develop a CEUS radiomics nomogram, and evaluate the role of a combined grey-scale ultrasound and CEUS model for the prediction and diagnosis of neoplastic GPs.

**Methods:**

Patients with GPs of ≥ 1 cm who underwent CEUS between January 2017 and May 2022 were retrospectively enrolled. Grey-scale ultrasound and arterial phase CEUS images of the largest section of the GPs were used for radiomics feature extraction. Features with good reproducibility in terms of intraclass correlation coefficient were selected. Grey-scale ultrasound and CEUS Rad-score models were first constructed using the Mann-Whitney U and LASSO regression test, and were subsequently included in the multivariable logistic regression analysis as independent factors for construction of the combined model.

**Results:**

A total of 229 patients were included in our study. Among them, 118 cholesterol polyps, 68 adenomas, 33 adenocarcinomas, 6 adenomyomatoses, and 4 inflammatory polyps were recorded. A total of 851 features were extracted from each patient. Following screening, 21 and 15 features were retained in the grey-scale and CEUS models, respectively. The combined model demonstrated AUCs of 0.88 (95% CI: 0.83 – 0.93) and 0.84 (95% CI: 0.74 – 0.93) in the training and testing set, respectively. When applied to the whole dataset, the combined model detected 111 of the 128 non-neoplastic GPs, decreasing the resection rate of non-neoplastic GPs to 13.3%.

**Conclusion:**

Our proposed combined model based on grey-scale ultrasound and CEUS radiomics features carries the potential as a non-invasive, radiation-free, and reproducible tool for the prediction and identification of neoplastic GPs. Our model may not only guide the treatment selection for GPs, but may also reduce the surgical burden of such patients.

## 1 Introduction

Gallbladder polyps (GPs) are common incidental findings on abdominal ultrasonography, with an incidence of approximately 5% among the adult population (1). GPs may be neoplastic or non-neoplastic (2). Current guidelines indicate surgical resection for neoplastic GPs of size ≥ 10 mm (3). However, the risk of unnecessary cholecystectomy has been reported, with only 20% and 7% of patients subjected to surgery shown to be diagnosed with adenomas, which possess malignant potential, and adenocarcinomas, respectively (4). Importantly, evidence have shown that surgery at the stage of carcinoma *in situ* can achieve a survival rate of 80% (5). As such, early diagnosis and treatment of neoplastic GPs remain vital for patient prognosis.

Abdominal ultrasonography is considered the imaging modality of choice for the evaluation of gallbladder lesions. However, conventional grey-scale ultrasound is limited in its ability to distinguish between neoplastic and non-neoplastic lesions. The introduction of contrast-enhanced ultrasonography (CEUS) addressed this by enabling the visualization of vascular patterns to lesions. However, the clinical role of CEUS in the field of gallbladder pathology remains debatable. Its efficacy for the differential diagnosis of benign and malignant GPs has been supported by several studies (6–8), with sensitivity and specificity of 87.1% and 69.0% reported, respectively (8). This is largely based on the ability of CEUS in characterizing the microcirculation of lesions (9). In contrast, other studies have reported the similarities in CEUS enhancement patterns between lesions (6, 7), alongside the technical difficulties with the imaging technique (7, 8), as well as the subjectivity in image interpretation resulting in substantial interobserver variations (9, 10).

Radiomics is a high-throughput quantitative image analysis technique involving the extraction of metrics invisible to the naked eye, and has greatly enhanced the diagnosis and prediction of malignant diseases (11–14). Studies on the use of ultrasound radiomics models for the classification of GPs currently exist (15–19), but some of them are limited in sample size (15, 16). In addition, the clinical value of a CEUS radiomics model has not been explored.

This study thereby aimed to develop a CEUS-based radiomics nomogram, and evaluate the role of a combined grey-scale ultrasound and CEUS model for the prediction and diagnosis of neoplastic GPs.

## 2 Methods

### 2.1 Patients

Patients with GPs detected on CEUS at our institute between January 2017 and May 2022 were retrospectively included. The inclusion criteria were as follows: (i) focal lesions ≥ 10 mm protruding into the gallbladder cavity; (ii) complete CEUS examination pre-operation; (iii) diagnosis confirmed on histopathology; and (iv) age ≥ 18 years. Adenomas and adenocarcinomas were defined as neoplastic GPs, while cholesterol polyps, adenomyomatosis, and inflammatory polyps were defined as non-neoplastic GPs.

### 2.2 The CEUS process

Ultrasound imaging was performed using either of the following regimens: Philips EPIQ 5 (Philips Medical System, Bothell, WA, USA) equipped with a C5-1 convex array probe (1.0 – 5.0 MHz), the pulse inversion imaging software, and mechanical index 0.04 – 0.08; or Acuson S3000 (Siemens Medical Solutions, Mountain View, CA, USA) equipped with a 6C1HD convex array probe (1.0 – 6.0 MHz), the contrast pulse sequencing/contrast high resolution imaging (CPS/CHI) software, and mechanical index 0.08 – 0.10. The contrast agent used was sulfur hexafluoride microbubble (SonoVue, Bracco, Milan, Italy) mixed in 5 mL saline. Following intravenous injection of 1.2 – 2.0 mL contrast agent through the antecubital vein, followed by a 5 mL flush of 0.9% sodium chloride solution, images were recorded for 120 s. After 60 s, the lesions were intermittently scanned and recorded for 5 min to evaluate for washout features. The grey scale ultrasound and CEUS exam were performed by JM.D. and HY. Z.(with 15 and 8 years’ experience of CEUS respectively). All images were saved and analyzed in a frame-by-frame approach.

### 2.3 Image segmentation and radiomics feature reproducibility assessment

**Figure 1:**
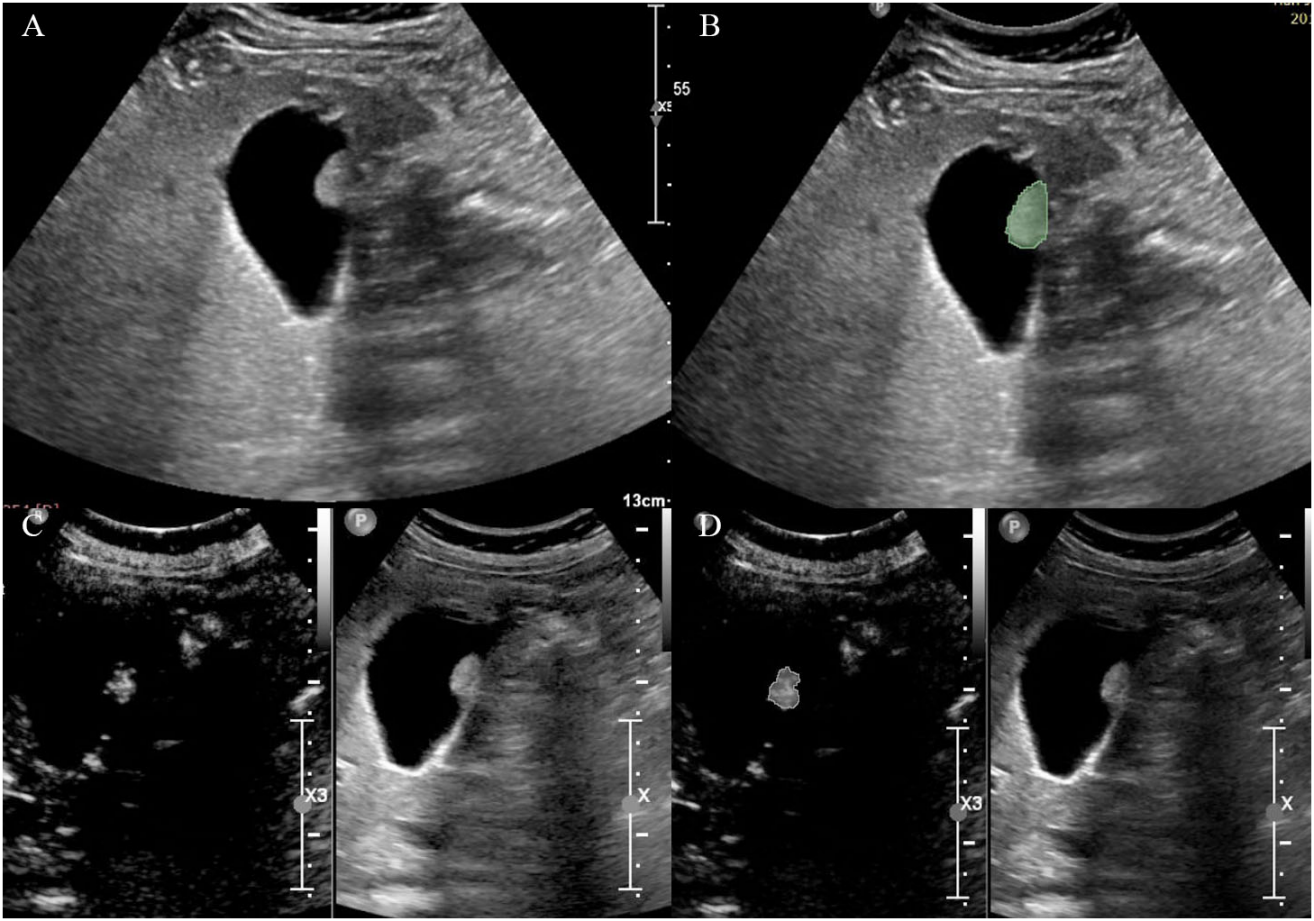
Delineation of the region of interest on **(A, B)** grey-scale ultrasound and **(C, D)** CEUS images.

Grey-scale ultrasound and arterial phase CEUS images of the largest section of the GPs were loaded into 3D-Slicer (\https://www.slicer.org). The region of interest (ROI) was defined as the boundary of the lesion, and was manually delineated by a sonographer (ZY.Q.). The delineation process is shown in **Figure 1**.

Interobserver agreement was analyzed using 50 randomly selected cases manually delineated by another sonographer (JM.D.). Intraclass correlation coefficient (ICC) of ≥ 0.75 indicated good reproducibility, and features with good reproducibility were retained for subsequent analysis.

### 2.4 Image preprocessing and radiomics feature extraction

Grey-scale ultrasound and CEUS radiomics features were extracted using PyRadiomics (\https://pyradiomics.readthedocs.io/). Data collected were as follows: first-order statistical features, two-dimensional morphological features, grey-level co-occurrence matrix (GLCM), grey-level size zone matrix (GLSZM), grey-level run-length matrix (GLRLM), grey-level dependence matrix (GLDM), and neighborhood grey tone difference matrix (NGTDM). In the presence of multiple polyps, the largest lesion was chosen for analysis.

### 2.5 Development and validation of the radiomics models

The cohort was divided into the training and testing set at a 7:3 ratio. For construction of the grey-scale ultrasound and CEUS radiomics model, radiomics features with ICC ≥ 0.75 were analyzed using the Mann–Whitney *U* test, and those of statistical significance were included in the least absolute shrinkage and selection operator (LASSO) regression analysis to select for features with non-zero coefficients. Penalty coefficient was adjusted based on 10-fold cross validation. The Rad-Score model is $\sum_{i=0}^{n}{\text{Coef}}_{i}\times X_{i}$ , where $Coef_{i}$ . is the risk coefficient of each feature in LASSO regression, and . the quantitative value of each feature. The two Rad-Score models were included in the multivariable logistic regression analysis as independent factors for construction of the combined model.

The predictive performance of all 3 models were assessed in terms of area under the receiver operating characteristic curve (AUC) and 95% confidence interval (CI), and were compared using the Delong test. The clinical utility of each nomogram was assessed in terms of net benefit under selected threshold probabilities using the decision curve analysis (DCA). Goodness of fit was evaluated using the Hosmer-Lemeshow test. Consistency between predicted and actual results was assessed using calibration curves.

All statistical analyses were performed using R v3.6.3 (R Foundation for Statistical Computing, Vienna, Austria). Statistical significance was defined as P< 0.05.

## 3 Results

### 3.1 Patient characteristics

A total of 229 patients were included in our study. Among them, 118 cholesterol polyps, 68 adenomas, 33 adenocarcinomas, 6 adenomyomatoses, and 4 inflammatory polyps were recorded. The baseline demographic and clinical characteristics of the included patients and GPs are shown in **Tables 1**, **2**, respectively.

**Table 1:** Baseline patient characteristics.

Characteristic		
Number of cases		229
Age (year)		50.9 ± 13.7 (20 – 77)
Sex		
Male		92
Female		137

**Table 2:** GPs characteristics.

Characteristic	
Number of GPs	229
Size (cm)	1.7 ± 0.9
Basal width	
<50% diameter	119
>50% diameter	110
Continuity of gallbladder wall	
Continuious	179
Discontinuious	50
Blood flow	
Present	85
Absent	144
Echoic feature	
Hyper	91
Iso	126
Hypo	12

### 3.2 Radiomics feature selection

**Figure 2:**
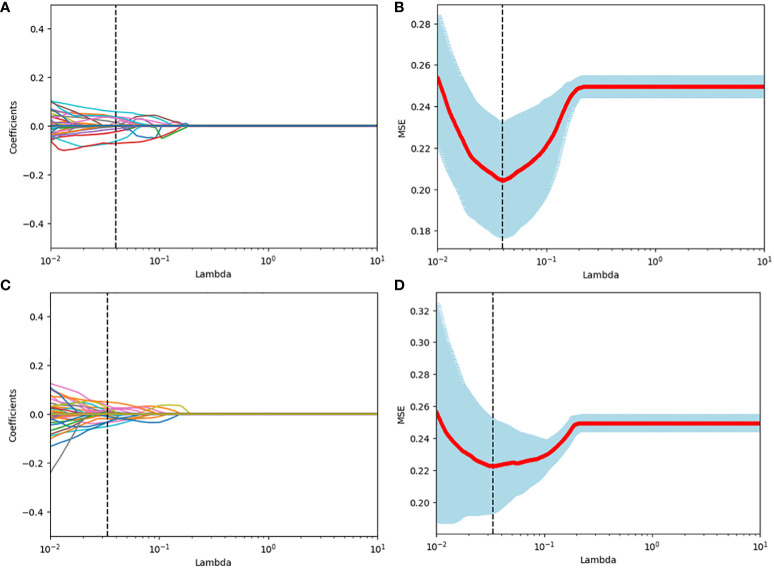
Radiomics feature selection using the LASSO binary logistic regression model. **(A, C)** LASSO coefficient profiles, displaying 309 and 391features. A coefficient profile plot was produced against the log (lambda) sequence. Each colored line represents the coefficient of an individual feature. A vertical line was drawn at the selected λ, where 5 features had non-zero coefficients. **(B, D)** Tuning parameter (log lambda) selection in the LASSO model using 10-fold cross-validation via minimum criteria. Vertical dotted lines were drawn at the selected λ values, corresponding to the chosen 5 variables that better fit the models.

**Figure 3:**
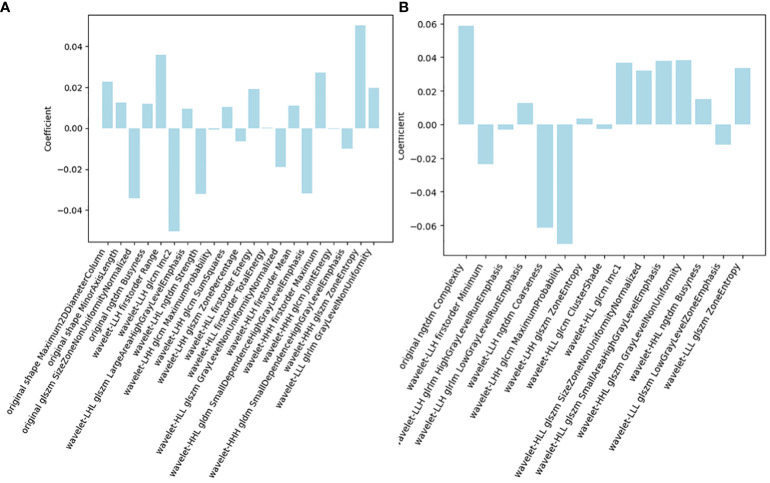
Radiomics features of the final **(A)** grey-scale ultrasound and **(B)** CEUS model.

Among the 851 radiomics features extracted from each patient, 715 grey-scale ultrasound and 755 CEUS features demonstrated good reproducibility. Following screening using the Mann-Whitney U and LASSO regression test, 21 and 15 features were retained, respectively, as shown in **Figure 2**. All grey-scale ultrasound and CEUS features included for subsequent analysis are shown in **Figure 3**; **Tables 3**, **4**.

**Table 3:** Radiomics features of the final grey-scale ultrasound model.

Characteristics	Coefficient
original shape Maximum2DDiameterColumn	0.000668581
original shape MinorAxisLength	0.000479755
original glszm SizeZoneNonUniformityNormalized	-0.919764882
original ngtdm Busyness	0.001722981
wavelet-LLH firstorder Range	1.4337×10^12^
wavelet-LLH glcm Imc2	-2.780125371
wavelet-LHL glszm LargeAreaHighGreyLevelEmphasis	3.34×10^-08^
wavelet-LHL ngtdm Strength	-0.550427027
wavelet-LHH glcm MaximumProbability	-0.021897724
wavelet-LHH glcm SumSquares	5.16807448
wavelet-LHH glszm ZonePercentage	-0.992930546
wavelet-HLL firstorder Energy	7.73×10^-07^
wavelet-HLL firstorder TotalEnergy	1.03×10^-08^
wavelet-HLL glszm GreyLevelNonUniformityNormalized	-0.230719978
wavelet-HLH firstorder Mean	3.20054×10^14^
wavelet-HHL gldm SmallDependenceHighGreyLevelEmphasis	-1.489858993
wavelet-HHH firstorder Maximum	7.1128×10^13^
wavelet-HHH glcm JointEnergy	-0.054269818
wavelet-HHH gldm SmallDependenceHighGreyLevelEmphasis	-0.871474888
wavelet-HHH glszm ZoneEntropy	0.12839212
wavelet-LLL glrlm GreyLevelNonUniformity	0.00012128209632913795

**Table 4:** Radiomics features of the final CEUS model.

Characteristic	Coefficient
original ngtdm Complexity	0.015737906
wavelet-LLH firstorder Minimum	-5.14374×10^12^
wavelet-LLH glrlm HighGreyLevelRunEmphasis	-0.016005186
wavelet-LLH glrlm LowGreyLevelRunEmphasis	0.277091907
wavelet-LLH ngtdm Coarseness	-0.662331938
wavelet-LHH glcm MaximumProbability	-1.714160955
wavelet-LHH glszm ZoneEntropy	0.007584999
wavelet-HLL glcm ClusterShade	-0.032961923
wavelet-HLL glcm Imc1	1.254993696
wavelet-HLL glszm SizeZoneNonUniformityNormalized	0.733963001
wavelet-HLL glszm SmallAreaHighGreyLevelEmphasis	0.025075348
wavelet-HHL glszm GreyLevelNonUniformity	0.011342027
wavelet-HHL ngtdm Busyness	2.55×10^-05^
wavelet-LLL glszm LowGreyLevelZoneEmphasis	-1.116617573
wavelet-LLL glszm ZoneEntropy	0.072778773

### 3.3 Predictive performance of the models

The grey-scale ultrasound radiomics model demonstrated an AUC of 0.82 (95% CI: 0.76 – 0.89), while the CEUS radiomics model showed an AUC of 0.86 (95% CI: 0.80 – 0.91). Both models were observed as independent predictors of neoplastic GP on multivariable logistic regression analysis (**Table 5**).

**Table 5:** Multivariate logistic regression analysis results of the models.

Characteristic	Multi Variable	
	*OR (95%CI)*	*P*
Rad-Score_greyscale	1.73×10^3^ (76.1 – 7.14 × 10^4^)	< 0.01
Rad-Score_CEUS	1.05 × 10^2^ (4.88 – 3.00 × 10^3^)	< 0.01

**Figure 4:**
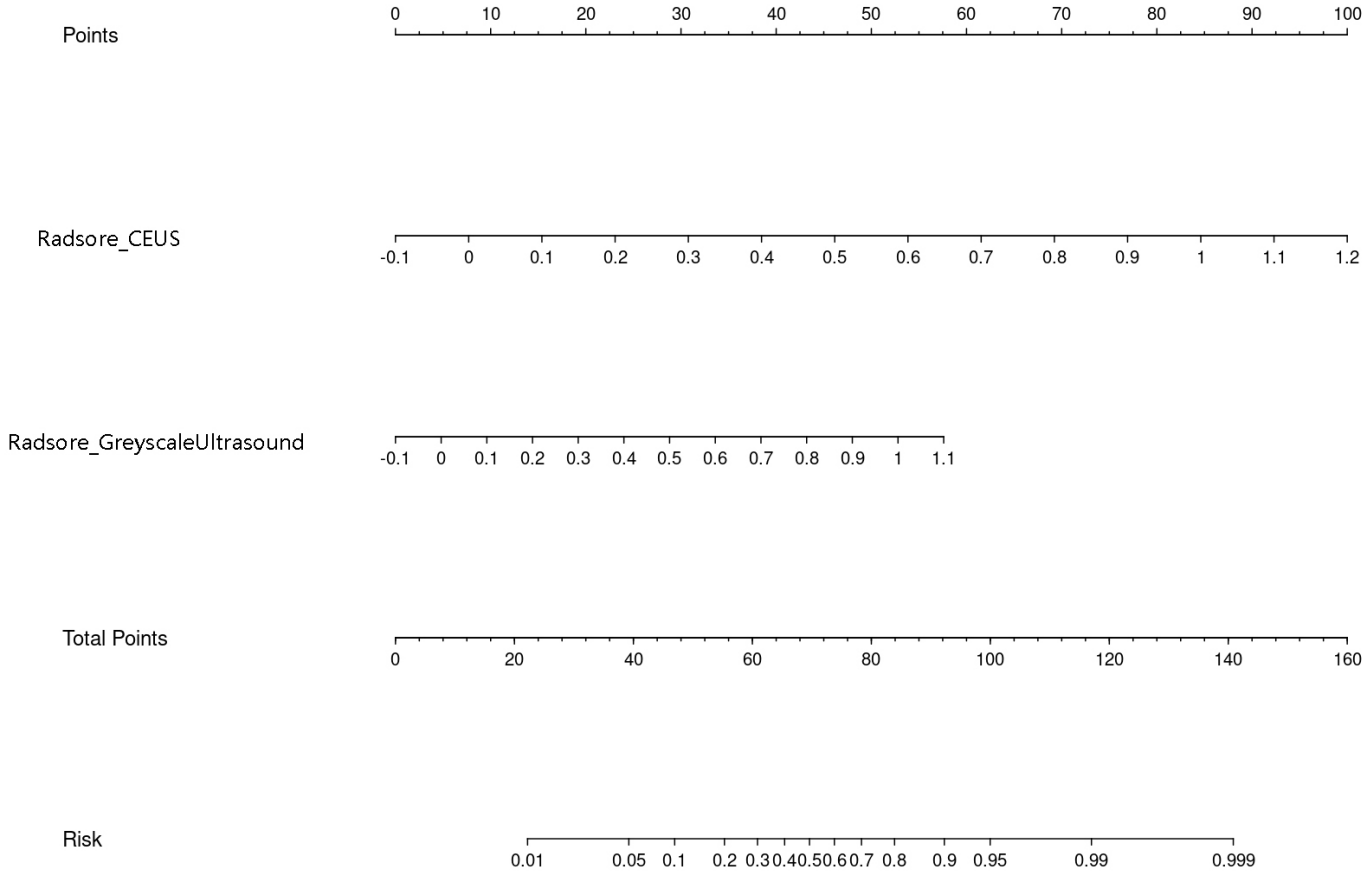
Nomogram of the combined radiomics model.

**Figure 5:**
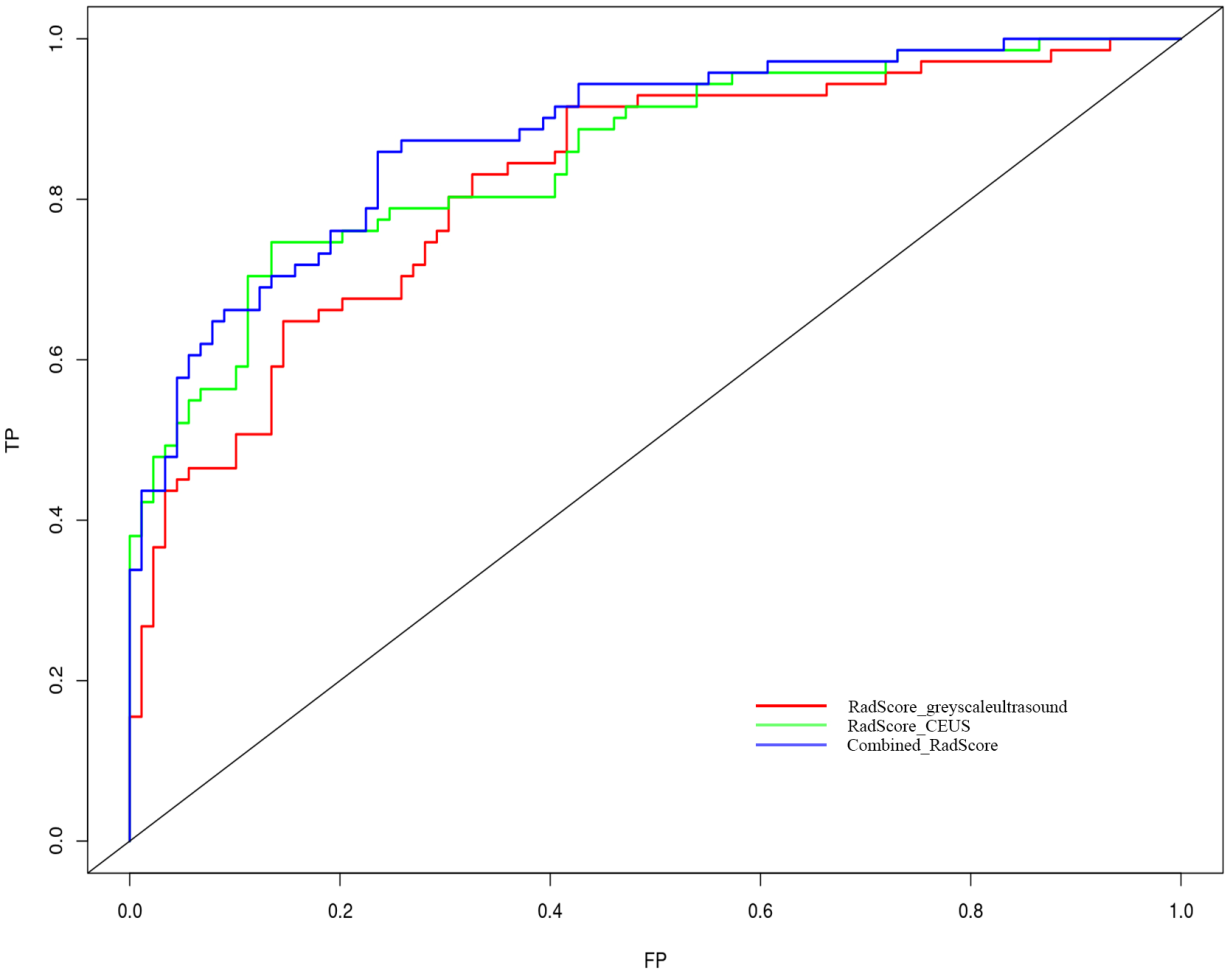
Receiver operating characteristic curves of the grey-scale CEUS and combined radiomics models. Grey-scale model *vs* CEUS model p=0.312; grey-scale model *vs* combine model p=0.010 and CEUS model *vs* combined model p=0.048.

**Figure 6:**
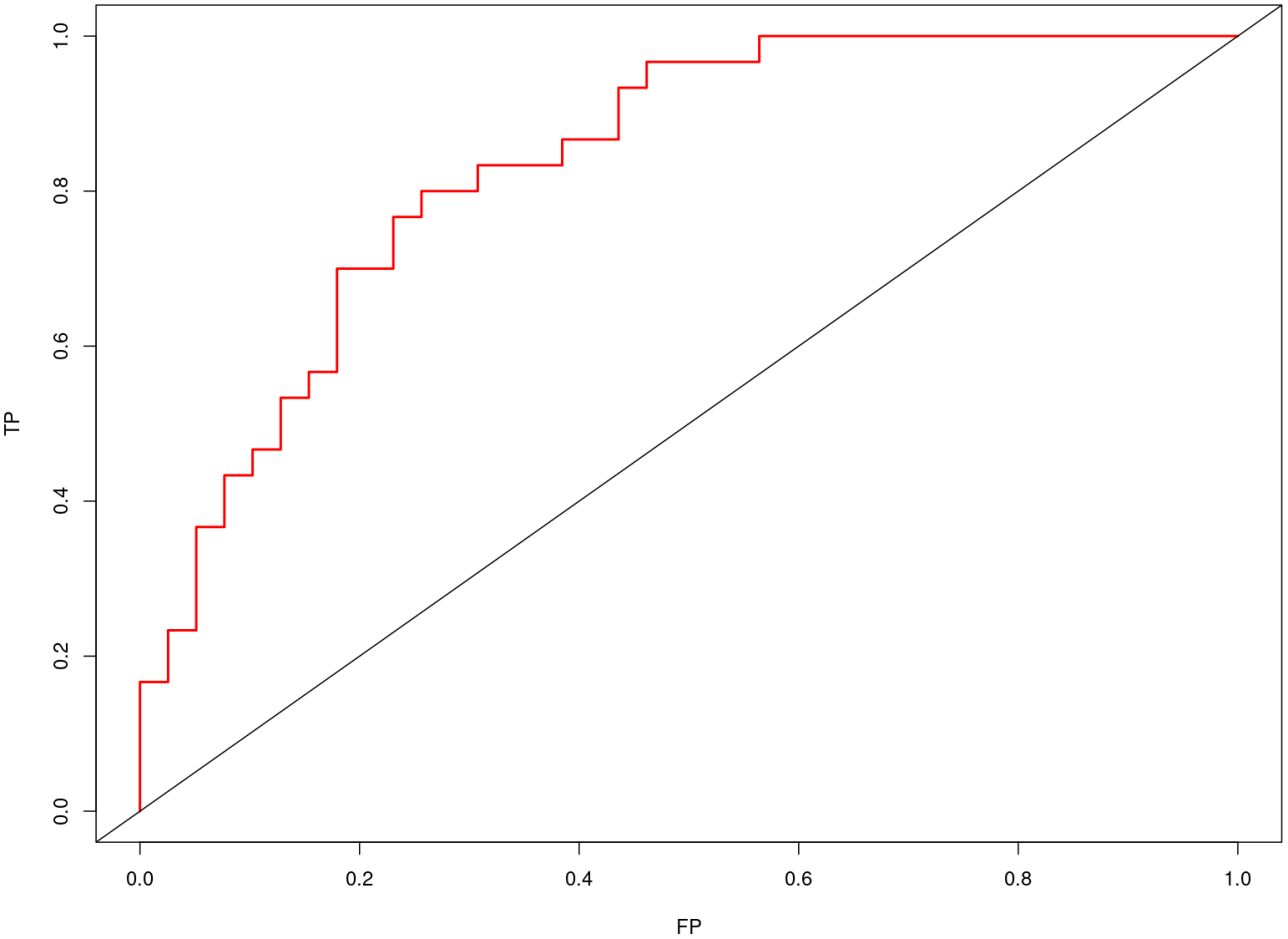
Receiver operating characteristic curves of the combined radiomics model in the testing set.

**Figure 7:**
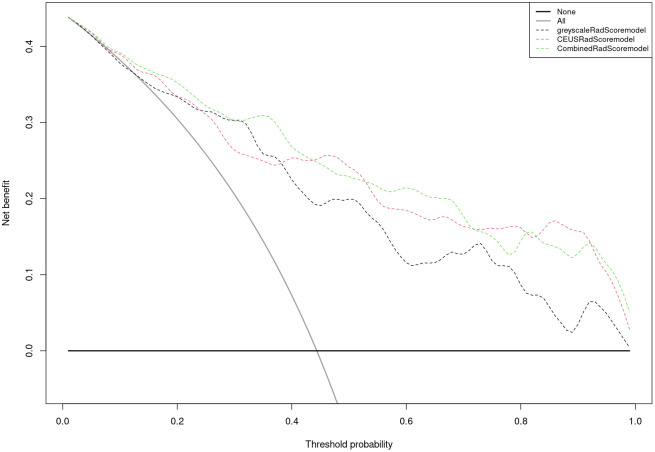
Decision curve analysis of the grey-scale, CEUS, and combined radiomics models.

**Figure 8:**
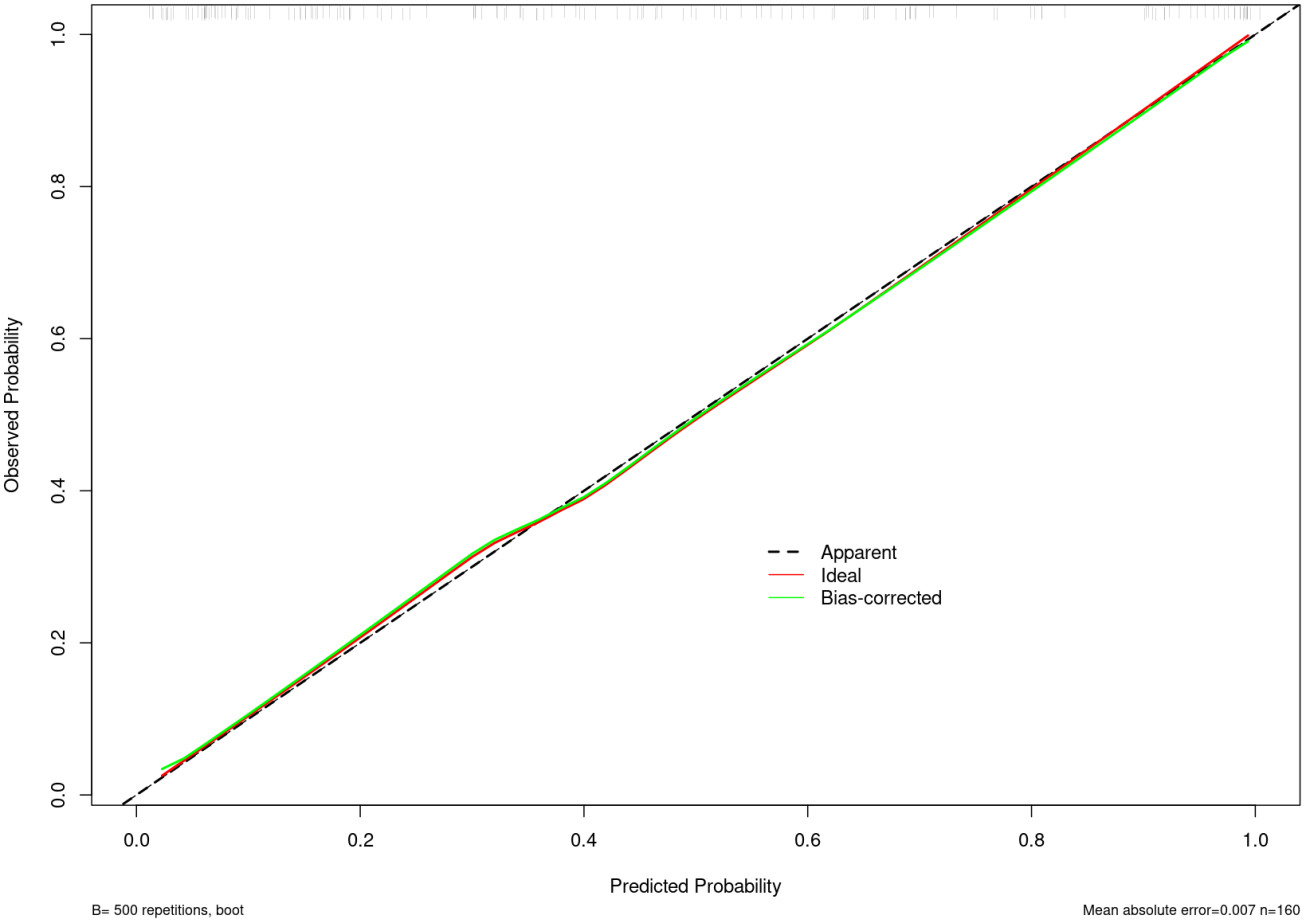
Calibration curve of the combined radiomics model in the training set.

Nomogram of the combined model is shown in **Figure 4**. AUCs of 0.88 (95% CI: 0.83 – 0.93) and 0.84 (95% CI: 0.74 – 0.93) were achieved in the training and testing set, respectively (**Figures 5**, **6**, respectively). And the combined model was observed to provide greater net benefits than the individual models at probability ranges of 0 – 0.43, 0.53 – 0.75, and > 0.91 on DCA (**Figure 7**). Good consistency between the predicted and actual results was further observed on calibration curve analysis (**Figure 8**).

### 3.4 Clinical application of the combined model

When applied to the whole dataset, the combined model demonstrated a sensitivity, specificity, and accuracy of 70.3% (95% CI: 60.4 – 79.0%), 87.5% (95% CI: 80.5 – 92.7%), and 80.0% (95% CI: 74.1 – 84.9%), respectively. The model detected 111 of the 128 non-neoplastic GPs, decreasing the resection rate of non-neoplastic GPs to 13.3%.

## 4 Discussion

Our proposed model combining the radiomics features of grey-scale ultrasound and CEUS showed good predictive performance and diagnostic accuracy for neoplastic GPs of ≥ 10 mm. Early surgical intervention has been shown to confer a prognostic benefit in neoplastic GPs. The five-year survival rate following gallbladder cancer surgery ranges between 2 – 80%, the 5-year survival rate can reach 80% for carcinomas in-situ. In the presence of lymph node metastasis, the 5-year survival rate drops to 2 – 8% in stage 4b gallbladder cancer (5). Surgery is indicated for neoplastic GPs of ≥ 10 mm based on current guidelines. However, challenges arise in distinguishing neoplastic from non-neoplastic GPs, given their similarities in imaging features, and that only a small proportion of GPs are neoplastic. The use of both grey-scale ultrasound and CEUS radiomics features was observed to enable a reduction in rate of non-neoplastic GPs resection to 13.3% in our study. As such, our proposed combined model carries the potential in minimizing unnecessary cholecystectomies among GPs of ≥ 10 mm. After CEUS, we could take the imaging data into the model. According to the result, if the GP is low risk of neoplastic polyp, just need follow-up until next CEUS or obviously larger in grey scale ultrasound. By applying this model, we may reduce the risk of surgery and the medical cost.

Conventional ultrasound represents the mainstay imaging modality for assessment of gallbladder lesions in terms of size, shape, and echogenicity. CEUS, a novel imaging modality, allows for visualization of the blood supply and vessel morphology of lesions, and has improved the detection of neoplastic GPs based on their high vascularity. The combined use of grey-scale ultrasound and CEUS can thereby enable the assessment of morphology, texture, and enhancement of GPs in an effective and objective manner (20). Following screening of radiomics features using the Mann-Whitney *U* test and LASSO regression with 10-fold cross validation, 21 and 15 features were eventually retained in the grey-scale and CEUS models, respectively. The grey-scale model included 2 shape-based features, 5 first-order features, and 14 texture features (4 GLCM, 1 GLRLM, 4 GLSZM, 5 NGDTM, and 2 GLDM), while the CEUS model included 1 first-order feature and 14 texture features (3 GLCM, 2 GLRLM, 6 GLSZM, and 3 NGDTM).

The grey-scale ultrasound-based first-order radiomics features retained in the model were Range, Maximum, Energy, Total Energy, and Mean. These features indicated that neoplastic GPs are larger in size, and are more heterogenous and hyperintense in echogenicity. Our final CEUS radiomics model further support the heterogenous nature of tissues in neoplastic GPs. In particular, the first-order feature, Minimum, which reflects the minimum enhancement intensity in the enhanced area, was observed to be significantly lower in neoplastic GPs. The remaining texture features in both models reflect space, distance, and other different aspects of information, which were complementary to the first-order features. Our findings revealed that higher Rad-Scores associated with the risk of neoplasm, reflecting the greater complexity in internal texture and distribution of neoplastic GPs, which may be because tumors of high malignant potential are usually characterized by large volume, high vascularity, and irregular shape (21). Importantly, by allowing for more comprehensive characterization of lesions, combination of the grey-scale and CEUS models was observed to achieve better predictive performance and clinical efficacy for the diagnosis of neoplasia.

Our study had several limitations. First, this study was retrospective in design, and limited in sample size, which may lead a problem of overfitting, so we used cross-validation and regularization method to improve the reliability of the model. We intend to collect more cases to conduct external verification to improve the reliability of the model in future study. Second, this study only included the GPs with pathology diagnosis. In addition, the use of different ultrasound devices and the lack of standardized image acquisition regimens may have affected the radiomics feature screening process. Lastly, the use of a dynamic and three-dimensional approach during feature extraction would have improved the predictive ability of our model.

## 5 Conclusion

Our proposed combined model based on grey-scale ultrasound and CEUS radiomics features carries the potential as a non-invasive, radiation-free, and reproducible tool for the prediction and identification of neoplastic GPs. Our model may not only guide the treatment selection for GPs, but may also reduce the surgical burden of such patients.

## Data availability statement

The raw data supporting the conclusions of this article will be made available by the authors, without undue reservation.

## Author contributions

ZQ: Conceptualization, Data curation, Formal analysis, Funding acquisition, Investigation, Methodology, Project administration, Resources, Software, Supervision, Validation, Visualization, Writing – original draft, Writing – review & editing. JD: Conceptualization, Data curation, Formal analysis, Funding acquisition, Investigation, Methodology, Project administration, Resources, Software, Supervision, Validation, Visualization, Writing – original draft, Writing – review & editing. YF: Conceptualization, Data curation, Formal analysis, Funding acquisition, Investigation, Methodology, Project administration, Resources, Software, Supervision, Validation, Visualization, Writing – review & editing. HZ: Conceptualization, Data curation, Formal analysis, Funding acquisition, Investigation, Methodology, Project administration, Resources, Software, Supervision, Validation, Visualization, Writing – review & editing. YW: Conceptualization, Data curation, Formal analysis, Funding acquisition, Investigation, Methodology, Project administration, Resources, Software, Supervision, Validation, Visualization, Writing – review & editing. XJ: Conceptualization, Data curation, Formal analysis, Funding acquisition, Investigation, Methodology, Project administration, Resources, Software, Supervision, Validation, Visualization, Writing – review & editing.
